# Factors that influence the satisfaction of people living with HIV with differentiated antiretroviral therapy delivery models in east Central Uganda: a cross-sectional study

**DOI:** 10.1186/s12913-023-09114-2

**Published:** 2023-02-08

**Authors:** Keith Baleeta, Augustin Muhwezi, Nathan Tumwesigye, Betty Nsangi Kintu, Sara Riese, Dathan Byonanebye, Martin Mbonye, Daniel Mwehire, Robert Iriso, Bernard Ayebazibwe, Lauren Bailey, Deborah Lopez, Laura McGough, Michael Etukoit, Dyogo Nantamu, Louisa Nakitende, Baker Tibengana, Jacob Wasswa

**Affiliations:** 1 University Research Co., LLC, Jinja, Uganda; 2 Family Health International 360, Kampala, Uganda; 3 College of Health Sciences, Makerere University, Kampala, Uganda; 4 United States Agency for International Development, Washington, DC USA; 5 The AIDS Support Organization, Kampala, Uganda; 6 Jinja District Local Government, Jinja, Uganda

**Keywords:** Differentiated service delivery model (DSDM), HIV care, Patient satisfaction, Community client-led ART delivery (CCLAD), Community drug distribution points (CDDP), Fast-track drug refill (FTDR)

## Abstract

**Background:**

The World Health Organization (WHO) and the Uganda Ministry of Health recommend differentiated service delivery models (DSDMs) as patient-centered antiretroviral therapy (ART) mechanisms for people living with HIV/AIDS (PLHIV) with undetectable viral loads. We studied patient satisfaction with ART services, and its associated factors amongst PLHIV enrolled in DSDMs in Uganda.

**Methods:**

This cross-sectional study involved a random sample of PLHIV accessing DSDM-related ART at nine facilities in East Central Uganda. Eligible patients were adult PLHIV (≥18 years), on ART, and enrolled for at least 12 months in one of three DSDMs: Community Client-Led ART Delivery (CCLAD), Community Drug Distribution Points (CDDP), or Fast-Track Drug Refill (FTDR). We collected data from June to July 2019. A validated tool measured satisfaction. General Estimating Equations with modified Poisson regression and exchangeable correlation structures accounted for clustering at health facilities and identified DSDM-related satisfaction factors.

**Results:**

Of 842 participants enrolled, 530 (63.5%) accessed HIV care through CDDP, 166 (20.1%) through CCLAD, and 146 (16.3%) through FTDR; 541 (64.2%) were satisfied with DSDM services: 78.7% in CDDP, 42.8% in CCLAD, and 36.3% in FTDR. The delivery and treatment factors positively associated with satisfaction included: being enrolled on CDDP [adjusted prevalence ratio (aPR) = 1.51, 95% CI:1.47–1.56] or FTDR [aPR = 1.47, 95% CI:1.26–1.71] relative to CCLAD and being enrolled in a DSDM for more than 3 years [aPR = 1.28, 95% CI:1.11–1.48]. Poor ART adherence [aPR = 0.33, 95% CI:0.19–0.56] and having a baseline WHO HIV stage of 3 or 4 [aPR = 0.36, 95% CI:0.20–0.64] relative to stages 1 and 2 were negatively associated. Among socioeconomic factors, having lower transport costs (< $1.35) per clinic visit [aPR = 1.34, 95% CI:1.17–1.53], being employed [aPR = 1.61, 95% CI:1.38–1.87], and being single [aPR = 1.10, 95% CI:1.08–1.13] were positively associated with satisfaction; drinking alcohol at least once a week [aPR = 0.77, 95% CI:0.63–0.93] was negatively associated with patient satisfaction.

**Conclusions:**

Results showed that 64.2% of patients were satisfied with DSDM services. HIV service delivery and treatment factors (DSDM type, time in DSDM, WHO stage, ART adherence), plus social factors (employment and marital status, transport costs, alcohol consumption), were associated with patient satisfaction. DSDM implementers should tailor services to address these factors to improve patient satisfaction.

**Supplementary Information:**

The online version contains supplementary material available at 10.1186/s12913-023-09114-2.

## Introduction

Since 2004, when free antiretroviral therapy (ART) became available in sub-Saharan Africa, remarkable progress has been made to accelerate ART initiation. An estimated 1.3–1.4 million Ugandans are living with HIV. Of those, 67% are on treatment, and 59.6% of adults in the reproductive age bracket (15–64 years) on ART are virally suppressed [1, 2]. More work remains to achieve the Joint United Nations Program on HIV and AIDS (UNAIDS) fast-track targets to achieve HIV epidemic control. The targets require that 95% of people living with HIV are aware of their status, 95% of those aware of their HIV-positive status are initiated on treatment, and 95% of those on treatment achieve and maintain viral suppression by 2030 [3].

To achieve these goals, care and treatment services must be responsive to the needs of individual patients and reduce barriers to care at each step of the HIV cascade [4]. To increase the responsiveness of ART programs, the World Health Organization (WHO) and funding agencies recommend the implementation of differentiated service delivery models (DSDMs) [3, 5]. Differentiated service delivery targets the “traditional public health response at a demographic group or geography in a manner that is responsive to the needs of the clients receiving care” [6]. In June 2017, building on the results of successful pilots, Uganda’s Ministry of Health released the “Implementation Guide for Differentiated Service Delivery Models of HIV Services in Uganda,” mandating that all facilities providing ART must implement DSDMs. However, the scale-up of this intervention (DSDM) was not implemented by Uganda’s Ministry of Health until 2019 [7].

The DSDMs recommended in Uganda for stable patients are:

- Fast-Track Drug Refill (FTDR): People living with HIV (PLHIV) pick up drugs from the ART dispensing points without being attended to by a clinician. However, the facility triage point checks in the patients for a quick assessment of urgent care complaints. Clinicians assess these patients once every 6 months to review adherence and determine eligibility for continuation in the FTDR program.
- Community Client-Led ART Delivery (CCLAD): PLHIV in care form groups within their communities and rotate drug pick-up from the facility or a community drug distribution point.
- Community Drug Distribution Points (CDDP): PLHIV pick up drugs and receive their clinical evaluations from community outreach points closest to them.
- Facility-Based Groups (FBGs): When stable PLHIV in care need peer support, these groups provide such support, as well as drug pick-up, as the leader of the group collects the drugs from the pharmacy for group members and distributes and accounts for them [7].

All DSDM approaches are designed to be responsive to patients’ needs in diverse ways and to reduce barriers to care to retain patients in care. Although there is limited evidence comparing DSDMs to traditional models, retention in care and viral suppression appear to be at least equivalent in DSDMs and traditional models [8]. One factor that may contribute to retention in treatment is patient satisfaction. Higher levels of patient satisfaction are associated with improved retention in HIV care and treatment [9, 10].

Non-DSDM HIV services in sub-Saharan Africa frequently report high satisfaction. Two studies from Nigeria, one in an HIV clinic in a tertiary hospital and another in smaller HIV clinics, reported high satisfaction with care in non-DSDM settings [11]. A study at a tertiary hospital in Cameroon reported 91% overall satisfaction with traditional HIV services [6]. Some studies from outside sub-Saharan Africa have reported lower patient satisfaction, below 50% [12].

Although patient satisfaction with DSDM has not been extensively studied, some studies show similarly high levels of satisfaction (80% or higher) with community-delivery models and facility-based groups in sub-Saharan Africa [13–15]. A recent mixed-methods study suggested that in Uganda, some ART patients may be more satisfied with standard clinic-based care while others appreciate community-based models [16].

Factors associated with patient satisfaction with HIV care, in general, can be observed at the individual and health-system levels. Individual-level factors associated with patient satisfaction with care include education level, expectation, and health status; the health system factors include accessibility, convenience and availability of services, and provider attitude and respectfulness [17].

DSDMs are designed to reduce barriers to HIV treatment, which may contribute to patient satisfaction. Whether an increase in patient satisfaction is achieved in practice in the Ugandan context, and whether certain DSDMs achieve higher patient satisfaction than others, is unknown. To our knowledge, no studies have measured and compared levels of satisfaction among patients enrolled in different DSDMs in Uganda. This study sought to determine the overall and domain-specific levels of satisfaction with care among HIV-infected patients receiving ART in DSDMs, as well as identify factors contributing to patient satisfaction among DSDMs, at nine health facilities in the East Central Region of Uganda.

## Methods

### Study design and setting

This cross-sectional study sought to determine patient satisfaction with care in adult HIV-infected patients enrolled in DSDMs at nine HIV facilities in six districts in East Central Uganda. The study facilities included two tertiary HIV care facilities primarily serving urban populations: The AIDS Support Organization (TASO) Jinja clinic and Jinja Regional Referral Hospital, and seven relatively high-volume facilities (Health Centre IVs and IIIs) that serve rural populations. All study facilities provide HIV prevention, testing, and treatment services with support from the U.S. President’s Emergency Plan for AIDS Relief (PEPFAR) through the Regional Health Integration to Enhance Services in East Central Uganda (RHITES-EC) project of the U.S. Agency for International Development (USAID). The facilities had implemented the WHO and Uganda Ministry of Health recommendations and had functional DSDMs.

Although each of the nine facilities had a relatively large HIV population (> 500 HIV patients active in care), there were critical differences in staffing and facility organization. These differences may influence client preferences or health facility delivery of one of the three DSDMs for stable clients. As of 2018, TASO Jinja had the highest (4047) number of patients enrolled in DSDMs for over 12 months. TASO Jinja clinic is a nongovernmental, specialized HIV treatment facility managed by TASO, Uganda. Jinja Regional Referral Hospital is a tertiary government hospital with a large, specialized ART clinic with 448 HIV patients enrolled in a DSDM for over 12 months, mostly in CCLAD and FTDR. The remaining facilities are public, with 500–1000 HIV-infected patients active in HIV care and fewer than 100 PLHIV in a DSDM for over a year.

These nine health facilities were selected because, at the time of the study’s data collection in 2019, they were the only health facilities in East Central Uganda with patients who had been enrolled in a DSDM for over 12 months. This is because these facilities had participated in the Ministry of Health DSDM pilot that preceded the 2019 country-wide scale-up of DSDMs.

### Study population, participants, and sampling

The study population comprised adult HIV-infected patients receiving ART from one of three DSDMs (CCLAD, CDDP, or FTDR) at the abovementioned nine study facilities within East Central Uganda. To be eligible for the study, a participant had to be HIV-positive, above 18 years of age and enrolled in one of three stable DSDMs (CCLAD, CDDP, or FTDR) at the nine study facilities within the East Central Region for at least 12 months. Also, they had to provide informed consent. Patients who had known psychiatric illnesses, and missing treatment records (particularly viral load results within the last 6 months and date of enrollment in a DSDM), were excluded from the study.

The Kish Leslie formula below was used to calculate the sample size for this study [18].

$${n}_0=\frac{Z^2\ast p\ast \left(1-p\right)}{e^2},$$

*Z* is the normal standard deviation at 95% confidence (1.96), *e*^*2*^ is the maximum acceptable error (*e* = 0.05), and *p* is the estimated patient satisfaction rate.

A previous cross-sectional study in rural clinics in Southwestern Uganda reported a patient satisfaction rate of 58% in patients attending outpatient HIV services [19]. Assuming a margin of error of 0.05, we estimated that the required sample size was 374 participants.

**Fig. 1:**
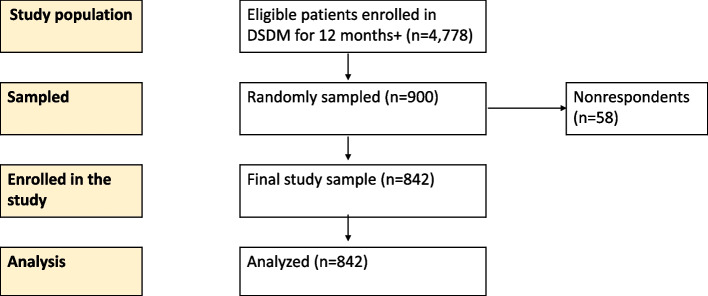
Flowchart illustrating participant recruitment into the study

We anticipated a nonresponse rate of 10%, and we utilized a design effect of two to account for individuals clustered at facilities. This design effect was referenced in a similar study in Malawi [20]. Accounting for these circumstances resulted in an adjusted sample size of 830. The final sample size was revised to 900 individuals to increase the rigor further. From a master list of all eligible patients (4778) selected from the ART registers from the nine facilities, a stratified random sample of 900 patients was determined. The patients were stratified based on patient facility population size while ensuring that, for representativeness, facilities with five or fewer patients in the final sampling frame had at least one patient sampled, as highlighted in Fig. 1 and the table in Additional file 1: Annex A.

### Ethical approval

We obtained ethical approval for the study protocol (TASOREC/014/19-UG-REC-009) from the accredited Review and Ethics Committee: The AIDS Support Organisation Review and Ethics Committee (TASO REC), whose registration number is UG-REC-009. The Uganda National Council of Science and Technology also granted regulatory clearance. This research study was purely observational (not experimental), with no removal of human tissue samples. All study participants were adults above 18 years of age. All eligible interview participants signed a written informed consent form before participating in the study: The study research assistant conducted the informed consent process before any study procedure. Consent was performed in as private a manner as possible. The study was fully explained to prospective study participants, and written informed consent was obtained. Prospective study participants were given adequate time to consider the information presented in the informed consent form. The consent form was signed and dated, and a copy was provided to each study participant. Informed consent was documented in writing on a consent form approved by the IRBs. An original English consent form was available for English-speaking study participants. For all other study participants, a Lusoga-translated version was used because Lusoga is the local language spoken by people in areas where the study clinics are located. A patient was free to withdraw from the study without penalty. Participants who could not read or write would undergo the consenting process in an impartial witness’s presence and would document approval with a thumbprint. The informed consent was read aloud in such cases. The study investigator or the investigator’s qualified designee reviewed the consent processes.

### Data collection

We used the UgandaEMR records of all patients registered for care at the study facilities. A database query was run to generate lists of all patients aged 18 years and older, on ART, and participating in DSDMs for at least 6 months. Charts of all prospective participants were retrieved and assessed for eligibility, data completeness (ART record and documented ART delivery model of any of the three DSDMs), and status of engagement determined (lost to follow-up versus active in care). For facilities that did not have an electronic patient record system, files of patients enrolled in DSDMs were retrieved manually and assessed for eligibility. From a line list of all eligible ART patients, a stratified random sample of patients by health facility was selected using Stata 14 (StataCorp, College Station, TX, USA).

Eligible patients were contacted to set an appointment for data collection. Patients in active care had interview appointments set for the most convenient location for the PLHIV, at the facility or in the community.

Trained research assistants administered the survey tool. A patient satisfaction survey forms captured data on sociodemographic and patient satisfaction domains. The questionnaire also captured data on treatment outcomes, including viral suppression from the patient ART card and the ART register.

### Development and validation of the patient satisfaction instrument

A review of patient satisfaction instruments for DSDMs for HIV care in program settings did not reveal any previously validated tools. Therefore, an adapted instrument was validated in this research. The initial items for the patient satisfaction instrument were generated during a brainstorming session with HIV service delivery experts in Uganda and from a qualitative literature review on patient experiences with DSDM.

The positive framing of patient satisfaction items can cause bias; therefore, items in the adapted instrument had a mix of positive and negative framing. Agree/disagree and yes/no response formats were also limited to reduce acquiescence bias [21].

The initial set of patient satisfaction items was pilot tested with a random sample of 30 patients at TASO Jinja and Jinja Regional Referral Hospital. Face validity of the interview questions was assessed using cognitive interviews with a subsample of 10 patients completing the pilot to confirm that the meaning of the questions was clear.

Patient satisfaction factors were extracted based on the reliability of the scale. Scale items were assessed for retention based on item-to-total (item-to-test) correlations and inter-item (item-to-rest) correlations. The reliability of the final scale was evaluated with Cronbach’s alpha coefficient. A corrected item-to-total correlation greater than 0.50 was considered adequate. Scale items with an average item-to-total correlation below 0.50 were deleted from the scale. A scale with a Cronbach’s alpha coefficient greater than 0.70 was considered to have an acceptable level of internal reliability [22].

The following items were thus retained with the following item-to-total correlations (see brackets): health worker confidentiality (0.73), psychosocial support received (0.84), time for other priorities (0.78), health cost (0.84), and time spent traveling and waiting to receive ART services (0.84). The resulting final scale of patient satisfaction, with five items and a Cronbach’s alpha of 0.82, was used to measure patient satisfaction with the different service delivery models tested in this study. Patients were then dichotomized as either satisfied or not satisfied.

In scoring satisfaction, a participant was considered satisfied if they were satisfied with all five items. One was “not satisfied” if they were not satisfied with any of the five items. For example, selecting “satisfied” with one out of the five items or four out of the five items on the scale was considered “not satisfied”.

### Data management and analysis

Each data collection form was assigned a unique ID. Completed survey questionnaires were reviewed for completeness and entered into an electronic online database daily. Open Data Kit was used for data entry. Prior to analysis, data were reviewed for completeness. Data were then exported from the study database to Stata 14 (StataCorp, College Station, TX, USA) for statistical analysis.

Descriptive statistics were computed and expressed as means (± standard deviations) for normally distributed continuous data or medians (interquartile range) for continuous but skewed variables. Categorical and ordinal data were summarized as proportions. Patient satisfaction was determined as a proportion of patients who reported satisfaction for all the validated domains of the patient satisfaction tool: health worker confidentiality, psychosocial support received, time for other priorities, health cost, and time spent traveling and waiting to receive ART services.

Unadjusted and adjusted prevalence ratios and their 95% CI were computed to determine associations between the covariates and patient satisfaction. The overall prevalence of patient satisfaction was over 10% (64.2%), so using odds ratios would overestimate the degree of association [23].

Accordingly, prevalence ratios were computed using General Estimating Equations with modified Poisson regression (i.e., Poisson regression with robust standard errors) and exchangeable correlation structures to account for clustering at health facilities. All factors significantly associated with patient satisfaction (*p* < 0.05) at the bi-variable analysis were included in the multivariable General Estimating Equations Poisson regression model [24–26].

## Results

### Sociodemographic characteristics of the respondents

Out of the 900 eligible study participants, a total of 842 respondents were enrolled in this study at nine healthcare facilities. Fifty-eight (58) eligible patients were nonrespondents either because they declined to participate or could not be readily traced.

Among the 842 patients, 588 (69.8%) were females. The median and interquartile range (IQR) age of the participants was 49 (42, 55). The youngest participant was 18 years old, while the oldest was 82. The study also showed that 486 (57.7%) had at least primary education, and 499 (59.3%) were married. The majority of the study participants (85.5%) were living below the poverty line (< $1.90/day). Furthermore, the study showed that 318 (71%) respondents resided less than 10 km from their nearest ART distribution point, more than half of the study participants (55.3%) had spent more than 10 years in HIV care, and 700 (83%) had spent more than 3 years in a DSDM, as shown in Table 1.

**Table 1:** Characteristics of study participants (*N* = 842)

Variable Name	Categories	FTDR (%)	CCLAD(%)	CDDP (%)	Totals (%)
Age (years)	18–29	10(8.3%)	7 (5.2%)	7 (1.5%)	24 (3.3%)
	30–39	30 (24.8%)	15 (11.1%)	56 (11.8%)	101 (13.8%)
	40–59	74(61.1%)	103 (76.3%)	381 (80.2%)	558 (76.3%)
	60+	7 (5.8%)	10 (7.4%)	31 (6.5%)	48 (6.6%)
Sex	Male	41 (28.1%)	56 (33.7%)	157 (29.6%)	254 (30.2%)
	Female	105 (71.9%)	110 (66.3%)	373 (70.4%)	588 (69.8%)
Period in HIV care (years)	< 10	90 (65.2%)	71 (43.3%)	208 (39.7%)	369 (44.7%)
	≥10	48 (34.8%)	93 (56.7%)	316 (60.3%)	457 (55.3%)
Period in DSDM (years)	< 3 years	98 (68.1%)	25 (15.1%)	16 (3.0%)	139 (16.6%)
	≥3 years	46 (38.9%)	141 (84.9%)	513(97.0%)	700 (83.4%)
Distance (km) to health facility	< 10	41 (59.4%)	48 (64.9%)	229 (75.1%)	318 (71.0%)
	≥10 km	28 (40.6%)	26 (35.1%)	76 (24.9%)	130 (29.0%)
Employment status	Unemployed	44 (31.2%)	47 (28.3%)	62 (11.7%)	15,318.3%)
	Employed	97 (68.8%)	119 (71.7%)	468 (88.3%)	684 (81.7%)
Average daily income	< $1.9	111 (78.7%)	139 (16.3%)	466 (87.9%)	720 85.5%)
	> $1.9	30 (21.3%)	27 (83.7%)	64(12.1%)	122 (14.5%)
Education	None	18 (12.8%)	12 (7.2%)	69 (13.0%)	99 (11.8%)
	Primary	51 (36.2%)	111 (66.9%)	322 (60.8%)	486 (57.7%)
	Secondary	60 (42.6%)	38 (22.9%)	177 (22.1%)	218 (25.9%)
	Tertiary	12 (8.5%)	5 (3.0%)	22 (4.1%)	39 (4.6%)
Marital status	Married	92 (65.3%)	109 (65.7%)	297 (56.1%)	498 (59.5%)
	Single	49 (34.7%)	57 (34.3%)	233 (43.9%)	339 (40.5%)
Drinking alcohol	Never	94 (66.7%)	133 (80.1%)	516 (97.4%)	745 (88.8%)
	At least once a week	47 (33.3%)	33 (19.9%)	14 (2.6%)	94 (11.2%)
Friend/relative at ART delivery point	No	81 (57.5%)	97 (58.4%)	206 (38.9%)	387 (46.0%)
	Yes	60 (42.5%)	69 (91.6%)	324 (61.1%)	455 (54.0%)

### Levels of satisfaction with DSDM services

Overall, 541 (64.2, 95% CI:59.2–69.2%) patients reported being satisfied with their DSDM: 78.7% in CDDP, 42.8% in CCLAD, and 36.3% in FTDR (Table 2).

**Table 2:** Level of satisfaction by DSDM type

DSDM type	N	Satisfied (*n* = 541)	Not satisfied (*n* = 301)
CCLAD	166	71 (42.8%)	95 (57.2%)
CDDP	530	417 (78.7%)	113 (21.3%)
FTDR	146	53 (36.3%)	93 (63.7%)
Overall	842	541 (64.2%)	301(35.8%)

As shown in Table 2, satisfaction levels varied considerably across the three DSDMs. Overall, patient satisfaction was lowest in patients enrolled in the facility-based DSDM (FTDR) and highest in CDDP.

### Correlates of patient satisfaction

The multivariate analysis revealed that the following factors were significantly associated with patient satisfaction with DSDM (*p* < 0.05), as described in Table 3.

**Table 3:** Multivariate analysis of correlates of patient satisfaction with DSDMs among patients enrolled for at least 12 months

Variable	Category	Patients on DSDM		Bivariate Analysis		Multivariate Analysis	
		Total (*N* = 842)	Satisfied n (%) (*N* = 541)	Unadjusted (Crude) Prevalence Ratio [95% CI]	*p*-value	aPR [95% CI]	*p*-value
DSDM	CCLAD	166	71 (42.8%)	Ref		Ref	
	CDDP	530	417 (78.7%)	1.98 [1.65–2.39]	< 0.001^a^	1.51 [1.47–1.56]	< 0.001^a^
	FTDR	146	53 (36.3%)	1.80 [1.47–2.20]	< 0.001^a^	1.47 [1.26–1.71]	< 0.001^a^
Gender	Female	588	366 (62.2%)	Ref		Ref	
	Male	254	175 (68.9%)	1.10 [1.03–1.16]	0.003^a^	1.10 [0.98–1.24]	0.106
Age category	18–29 years	24	12 (50.0%)	Ref			
	30–39 years	101	70 (69.3%)	1.20 [0.95–1.53]	0.125		
	40–59 years	560	391 (69.8%)	1.13 [0.91–1.41]	0.28		
	≥60 years	48	33 (68.8%)	1.10 [0.98–1.23]	0.092		
Period in HIV care (Years)	< 10 Years	369	232 (62.9%)	Ref		Ref	
	≥10 Years	457	296 (64.8%)	0.91 [0.90–0.93]	< 0.001^a^	1.00 [0.96–1.03]	0.923
Period in DSDM (Years)	1–2 years	139	46 (33.1%)	Ref		Ref	
	3+ years	700	494 (70.6%)	1.44 [1.08–1.92]	0.014^a^	1.28 [1.11–1.48]	0.001^a^
Poor adherence to ART (Last 3 days)	No	732	523 (71.5%)	Ref			
	Yes	110	18 (16.4%)	0.57 [0.48–0.67]	< 0.001^a^	0.33 [0.19–0.56]	< 0.001^a^
Baseline WHO stage	Stage 1 or 2	796	532 (66.8%)	Ref		Ref	
	Stage 3 or 4	35	7 (20.0%)	0.49 [0.33–0.73]	< 0.001^a^	0.36 [0.20–0.64]	< 0.001^a^
HIV viral load suppression	Detectable HIV viral load	130	79 (60.8%)	Ref			
	Undetectable HIV viral load	666	436 (65.5%)	0.95 [0.82–1.09]	0.857		
Employment status	Unemployed	153	50 (32.6%)	Ref		Ref	
	Employed	684	491 (71.8%)	2.00 [1.73–2.31]	< 0.001^a^	1.61 [1.38–1.87]	< 0.001^a^
Average income/day	< $1.90/ day	720	470 (65.3%)	Ref	Ref		
	> $1.90/ day	122	71 (58.2%)	1.02 [0.98–1.06]	0.395		
Education level	No education	99	66 (66.7%)	Ref			
	Primary education	486	324 (66.7%)	1.02 [0.93–1.12]	0.687		
	Secondary education	218	129 (59.2%)	1.05 [0.99–1.12]	0.069		
	Tertiary education	39	22 (56.4%)	1.02 [0.82–1.29]	0.835		
Marital status	Married	499	307 (61.5%)	Ref		Ref	
	Single	114	81 (71.1%)	1.12 [1.07–1.18]	< 0.001^a^	1.10 [1.08–1.13]	< 0.001^a^
Transport costs to facility	≥5000 UGX ($1.35)	231	106 (45.9%)	Ref	Ref		
	< 5000 UGX ($1.35)	611	435 (71.2%)	1.39 [1.32–1.46]	< 0.001^a^	1.34 [1.17–1.53]	< 0.001^a^
Estimated distance to health facility	< 10 KM	318	249 (78.3%)	Ref		Ref	
	≥10KM	130	72 (55.4%)	0.71 [0.66–0.76]	< 0.001^a^	0.98 [0.87–1.10]	0.735
Friend/relative at ART point	No	387	206 (53.2%)	Ref	Ref	Ref	
	Yes	455	335 (73.6%)	1.36 [1.24–1.49]	< 0.001^a^	1.01 [0.99–1.02]	0.292
Alcohol consumption	Fewer than once a week	745	517 (69.4%)	Ref	Ref		
	At least once a week	97	24 (24.7%)	0.55 [0.36–0.85]	0.008^a^	0.77 [0.63–0.93]	0.008^a^

^a^ significant variables at bivariate and multivariate analysis

Among the socioeconomic and demographic factors, patients who incurred lower transport costs (< $1.35) per clinic visit [adjusted prevalence ratio (aPR) = 1.34, 95% CI:1.17–1.53] had a prevalence of patient satisfaction that was 1.34 times greater than those with higher costs per clinic visit. In addition, patients who were employed [aPR = 1.61, 95% CI:1.38–1.87] had a 1.61 times greater prevalence of patient satisfaction than those who were unemployed. It was also noted that patients who drank alcohol at least once a week [aPR = 0.77, 95% CI:0.63–0.93] had a 23% lower prevalence of satisfaction than those who never drank alcohol. Similarly, patients on DSDM who were single (i.e., no marital partner) had a slightly higher prevalence of patient satisfaction [aPR = 1.10, 95% CI:1.08–1.13] relative to patients on DSDM who were married.

Regarding service delivery and treatment-related factors, patients enrolled in CDDP [aPR = 1.51, 95% CI:1.47–1.56] and FTDR [aPR = 1.47, 95% CI:1.26–1.71] had a significantly greater prevalence of patient satisfaction relative to patients on CCLAD. Regarding the duration of time on a DSDM, patients who had greater than 3 years on a DSDM [aPR = 1.28, 95% CI:1.11–1.48] had a 1.28 times greater prevalence of patient satisfaction than patients who had spent less than 3 years on a DSDM. In addition, patients who poorly adhered to ART during the 3 days before the study interview [aPR = 0.33, 95% CI:0.19–0.56] had a 67% lower prevalence of patient satisfaction than patients with good ART adherence in the 3 days preceding the study interview. Finally, patients with a baseline DSDM enrollment WHO stage of 3 or 4 had a lower prevalence of patient satisfaction [aPR = 0.36, 95% CI:0.20–0.64] than patients with a baseline of stage 1 or 2. No significant association was found between patient satisfaction and HIV viral load suppression [aPR = 0.95, 95% CI:0.82–1.09].

## Discussion

Patient satisfaction is a commonly used, critical indicator in evaluating healthcare service quality [27]. This study sought to fill gaps in knowledge around patient satisfaction for those enrolled in DSDMs of HIV care for stable patients in East Central Uganda [12].

### Levels of satisfaction with DSDM services

In this study, 64.2% of participants were satisfied with services in their DSDM, which is high. We did not find similar studies assessing patient satisfaction within a DSDM. Still, compared to studies done assessing patient satisfaction within routine HIV care, the level of satisfaction with this study’s subject DSDMs was within the same range as regular HIV services, which extended from 44 to 95%. It is important to note that this large variability of patient satisfaction between the various studies may be attributed to differences in patient satisfaction scales and cutoff levels used in patient satisfaction assessments. This large variability is thus a limitation as it makes it challenging to get narrower confidence intervals of the level of patient satisfaction from the literature review [12, 27].

Regarding the relationship between the DSD model type and patient satisfaction, this study found that enrollment in the CDDP and FTDR DSDMs was significantly associated with patient satisfaction after controlling for other patient-level factors. And overall, PLHIV in CDDP had the highest level of satisfaction. Possible explanations for these associations may be attributed to the socio-demographic differences among individuals enrolled in these different DSDMs. For instance, Table 1, which provides descriptive statistics stratified by DSDM, PLHIV registered in CDDP significantly differed from those in CCLAD and FTDR; the former had larger proportions of individuals in the categories significantly associated with patient satisfaction. For example, more individuals had spent longer in HIV care (> 10 years), longer in the DSDM (3 years), were employed, and did not drink alcohol.

A systematic review of several studies of DSDMs in sub-Saharan Africa identified that urban populations preferred care via a facility-based DSDM (e.g., FTDR); in contrast, rural people favored community-based drug collection (e.g., CDDP) [28]. Our study did not explicitly assess how an urban versus rural residence impacted the study participants’ satisfaction, which is a limitation. Hence, dwelling in an urban or rural setting may be a residual confounding factor not explicitly assessed in this study.

Two critical aspects of a patient’s HIV history were found to be significantly negatively associated with satisfaction with their DSDM after controlling for other patient-level factors: poor adherence and a baseline WHO stage of 3 or 4. Similar findings have been documented in numerous studies carried out among patients receiving routine HIV service delivery, where good ART adherence was positively associated with patient satisfaction. Several other studies have found statistically significant associations between patient satisfaction and intensified patient psycho-social support at the ART service delivery point focused on improving patient adherence [9, 29–31].

In routine HIV care, findings from Ethiopia and Vietnam have shown similar associations to what was observed in our study, i.e., In these two studies, patient satisfaction was positively associated with a higher CD4 count and lower HIV stage. Furthermore, higher satisfaction was also associated with a better quality of life and reduced illness episodes for individuals with early HIV disease [32, 33].

The lack of significant association between viral load suppression and patient satisfaction is similar to findings reported in several other studies from sub-Saharan Africa [9, 29].

The finding [34] that a more extended period of enrollment in a DSDM (greater than 3 years) was significantly associated with patient satisfaction can be attributed to reduced clinic visits, reduced transport costs, and enhanced psychosocial support in the DSDM [18]. This association of longer duration in DSDM may also be attributed to those with higher patient satisfaction staying in HIV care longer. Several studies in routine HIV care done in Canada, Vietnam, and Cameroon have found no significant association between the duration of ART and patient satisfaction or between the date since HIV diagnosis and patient satisfaction [12, 17, 35]. Further research, including longitudinal studies of patients enrolled in DSDMs, would help determine the relationship between the service delivery model, patient satisfaction, and retention in care in DSDMs.

In this study, PLHIV, who spent less than $1.35 on transport per clinic visit, demonstrated increased patient satisfaction relative to those who spent more than $1.35 on transportation at each clinic visit. These findings are in line with several research studies and systematic reviews done from patients enrolled in DSDMs, where reduced transport costs are identified as one of the key benefits of participation in a DSDM, especially one of the community DSDMs [36, 37]. It is also important to note that our patient satisfaction measures included an item about the time spent traveling to receive ART services. Patients with lower transport costs may be more likely to have shorter travel times to ART services, leading to higher satisfaction levels with that item.

While our study found a significant relationship between being employed and patient satisfaction, previous evidence from other countries in sub-Saharan Africa has been mixed [12, 34].

In this study, 11.2% of study participants reported alcohol intake at least once a week; however, not drinking alcohol was significantly associated with patient satisfaction for patients in a DSDM. There are no other documented studies assessing the relationship between patient satisfaction and alcohol consumption for patients in a DSDM [16].

It is important to note that each of the referenced studies in this discussion used different satisfaction assessment measures, contributing to some of the discrepancies in the findings. Specifically, our assessment tool for patient satisfaction required patients to report being satisfied with all five items in the measure.

### Study strengths and limitations

This study provides valuable information on patient satisfaction with DSDMs for HIV treatment in East Central Uganda. The sample size was large enough to detect significant differences between the groups. We also developed a specific patient satisfaction measure designed for patients enrolled in DSDMs.

Despite these strengths, this study also had limitations, as all studies do. First, we used a cross-sectional study design that does not allow for inference for causality since temporality cannot be established. As illustrated earlier in the discussion of patient satisfaction and length of time in a DSDM or treatment adherence, the associations observed may be driven by the independent variable affecting the dependent variable, or it may be the dependent variable driving the association with the independent variable. Second, we depended on self-reported responses that could be affected by recall or courtesy bias. Courtesy bias may have artificially inflated the estimates of positively perceived items, such as satisfaction and adherence, and artificially lowered the estimates of items viewed negatively, such as alcohol use. Third, there may have been nonresponse bias as not all participants invited for the interview were available for the face-to-face interview; there is a possibility that they were dissatisfied with the services provided at the ART service delivery point. Finally, only including PLHIV active in care on a DSDM may have introduced selection bias, as dissatisfied PLHIV may have dropped out of HIV care. These last two limitations could have led to artificially inflated patient satisfaction estimates in this study.

## Conclusions

The study showed that 64.2% of the patients who participated were satisfied with services in DSDMs. In both bivariate and multivariate analyses, patients in CDDP were more satisfied with their DSDM than patients in CCLAD or FTDR. Certain aspects of a patient’s clinical history and sociodemographic background are also factors in their satisfaction. Implementers of DSDMs need to tailor services to address these factors to improve patient satisfaction, especially in CCLAD and FTDR DSDMs. Such improvements will be critical to enhance HIV patient health outcomes, including adherence to ART and retention in HIV care services in Uganda and sub-Saharan Africa.

## Supplementary Information

**Additional file 1: Annex A.** Sampling of patients from the study sites with details on study population and final sample sizes.

## Data Availability

The datasets used during this study are available from the corresponding author on request.

## Abbreviations

aPR: Adjusted Prevalence Ratio
ART: Antiretroviral therapy
CCLAD: Community Client-Led ART Delivery
CDDP: Community Drug Distribution Points
DSDM: Differentiated Service Delivery Model
FTDR: Fast-Track Drug Refill
PEPFAR: U.S. Presidents’ Emergency Plan for AIDS Relief
PLHIV: People Living with HIV
TASO: The AIDS Support Organization
UNAIDS: Joint United Nations Program on HIV and AIDS
URC: University Research Co., LLC
USAID: U.S. Agency for International Development
WHO: World Health Organization
