# Supplementary material for: Factors that influence the satisfaction of people living with HIV with differentiated antiretroviral therapy delivery models in east Central Uganda: a cross-sectional study
Source: BMC Health Serv Res. 2023 Feb 8;23:127. doi: 10.1186/s12913-023-09114-2 (PMC9906920; doi:10.1186/s12913-023-09114-2)
Supplement: Supplementary file 1 — Additional file 1: Annex A. Sampling of patients from the study sites with details on study population and final sample sizes. [file 12913_2023_9114_MOESM1_ESM.docx]

**Annex A**

**Sampling of patients from the study sites with details on study population and final sample sizes.**

| Health Facility | Sample population | Final Sample |
| --- | --- | --- |
| Banda HCIV | 37 | 4 |
| Bugono HC IV | 3 | 3 |
| Busesa HC IV | 1 | 1 |
| Busia HC IV | 2 | 2 |
| Jinja RRH | 448 | 87 |
| Kigandalo HCIV | 5 | 1 |
| Kityerera HCIV | 74 | 8 |
| TASO Special Clinic | 4079 | 715 |
| Wabulungu HCIII | 129 | 21 |
| Grand Total | 4778 | 842 |
